# Variability in Forest Visit Numbers in Different Regions and Population Segments before and during the COVID-19 Pandemic

**DOI:** 10.3390/ijerph18073469

**Published:** 2021-03-26

**Authors:** Magdaléna Pichlerová, Dilek Önkal, Anthony Bartlett, Jozef Výbošťok, Viliam Pichler

**Affiliations:** 1 Faculty of Ecology and Environmental Sciences, Technical University in Zvolen, T. G. Masaryka 24, 960 01 Zvolen, Slovakia; magdalena.pichlerova@tuzvo.sk; 2 Department of Marketing, Operations and Systems, Newcastle Business School, Northumbria University, Newcastle upon Tyne NE1 8ST, UK; dilek.onkal@northumbria.ac.uk; 3 Tacit Bio, Bennett Corner House, 33 Coleshill Street, Sutton Coldfield B72 1SD, UK; tonybartlett@compuserve.com; 4 Faculty of Forestry, Technical University in Zvolen, T. G. Masaryka 24, 960 01 Zvolen, Slovakia; jozef.vybostok@tuzvo.sk

**Keywords:** forest recreation, forest coverage, settlement size, COVID-19 pandemic, lockdown restrictions, number of forest visits, forest visitor age

## Abstract

In view of the prevailing preferences for health and recreation revealed by previous studies as the main expected benefits of forest visits, the research presented herein focuses on whether such expectations would translate into a significant increase in the number of forest visits (NFV) following pandemic outbreaks. In this context, a Slovak nationwide survey on forests was conducted, with the main objective of casting light on possible changes in NFV as a coping mechanism or behavioral response to the discomfort and severe restrictions stemming from coronavirus disease 2019 (COVID-19) and the related measures. The survey was administered on a statistically representative sample after the pandemic’s first wave ebbed and restrictions were eased in the summer months of 2020. Collected data were assessed using ANOVA, the results of which supported the importance of forests as places providing opportunities for restoration of mental and physical resources. Forest accessibility as represented by forest coverage and settlement size emerged as a paramount factor affecting NFV rates both before and during the COVID-19 pandemic. The pandemic and its accompanying measures affected the relationships between NFV and average per capita income, type of employment, and most importantly age, highlighting possible vulnerabilities and disadvantages in certain population segments.

## 1. Introduction

The social value of forest recreation mainly relates to intangible benefits, such as aesthetic qualities, as well as the enhancement of psychological and physical health [1]. The results of a considerable amount of research have shown that nature-based outdoor activities, forest visits, and environmental simulation promote both physical and mental health and wellbeing, leading to more positive emotional self-reports and increased sustained attention, cognitive functions, and processes of restoration. Further reported benefits include physiological stress reductions manifested by various indicators, e.g., improvements in heartbeat and heart rate variability, blood pressure, stress hormone levels, and others [2,3,4,5,6]. Since the 1990s, outdoor and forest recreation has been on the political agenda at the European level [7]. However, contradictions exist between the expressed political importance of outdoor recreation at the national level and the absence of binding commitments for action [8]. One reason for this could be that the role of forest management in contributing to “health bonuses” in the form of human and public health benefits provided by forests is still poorly investigated [9]. The components of professional forestry may consist of selecting appropriate tree species, supporting forest stand resilience, and maintaining aesthetically pleasing forests, among other activities. 

Discussions and policies on forest recreation and management need to be reviewed in light of the multidimensional impacts of the coronavirus disease 2019 (COVID-19) pandemic, caused by a highly contagious virus that rapidly spreads and continuously evolves with new variants [10]. Uncertainty surrounding vaccine availability and uptake, along with the fact that this virus rapidly circulates throughout the world and mutates, has led to a strong focus on a variety of public health measures, e.g., quarantine, lockdowns, and social distancing [11]. The coronavirus outbreak has also profoundly impacted the delivery of essential healthcare and management practices in core clinical settings across the globe, including health rationing, which has affected many lives [12]. The overall cost of health risks due to the COVID-19 pandemic is exceptionally high [13]. Historical analogies reveal that such situations may lead to long-term issues in accessing healthcare and rising costs for medical treatments, in turn producing a growing demand for alternative ways of coping with health problems and lack of wellbeing [14]. Along similar lines, research into SARS-CoV-2 (hereinafter referred to as COVID-19) infection has shown that recovery is commonly affected by persistent fatigue, accompanied by anxiety or depression and other symptoms [15], thus pointing to multifaceted health repercussions and individuals’ search for alternative methods of restoring mental and physical wellbeing. 

Nature and forest visits have various restorative effects on health, including stress reduction, whereby the feelings of restoration, vitality, and positive mood increase, with older forests showing significantly stronger effects compared to young forests [16,17]. Additionally, spending time in nature and forests appears to produce health benefits through enhanced immune functioning, promoted by various compounds and microorganisms contained in the forest air [5]. These findings make the forests–public health link profoundly relevant in the current pandemic situation. The complex consequences of the COVID-19 pandemic, along with its accompanying measures (e.g., limitations on accessing health services, lockdown, social distancing) and side-effects (e.g., loneliness, anxiety, depression, weight gain, increased alcohol consumption, reduced physical activity), are likely to last for an uncertain length of time into the future [18,19]. This necessitates public health initiatives and creative solutions to various wellbeing and social challenges as the global pandemic takes its heavy toll. Given the restorative effects mentioned above, our study aims to examine whether people seek relief through increased forest visits and recreation to cope with the difficulties and uncertainties of the pandemic. Our working hypothesis is that the number of forest visits (NFV) would increase during the COVID-19 pandemic as compared to the pre-pandemic period, and this would especially be the case for people with easy access to forests. The hypothesis drew on our 2018 survey that indicated high preference for forest recreation compared to other forest ecosystem services, such as wood or biomass production [20]. We expected this tendency to be reinforced in the face of the pandemic restrictions, and therefore analyzed the number of forest visits in Slovakia—a country well-known for its collection of vast forests that cover approximately 40% of its total area.

## 2. Materials and Methods

In order to establish the anticipated change in the number of forest visits during the early phase of the COVID-19 pandemic, we conducted a nation-wide survey on a representative sample of respondents. Considering that the population size for each stratum was known, the sample size for each stratum was determined using the Krejcie and Morgan formula [21]. The required sample sizes, along with the realized sample sizes, are shown in Table 1. The realized sample size in most cases reached or exceeded the required sample size, except for respondents in the age category of 16–29 and residents of Bratislava, the capital of Slovakia.

**Figure 1:**
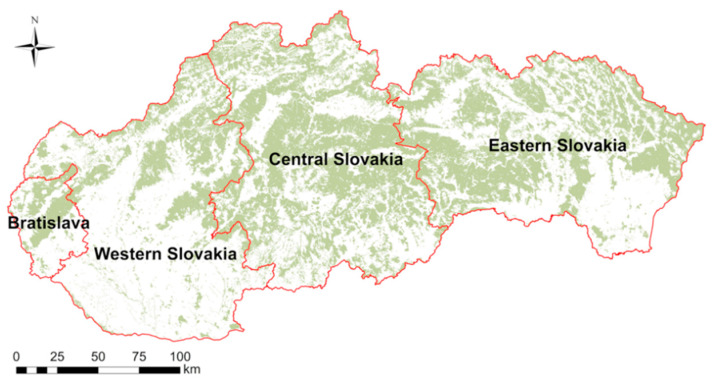
Forest coverage in geographical regions of Slovakia: Bratislava (3.7%), Western Slovakia (19%), Central Slovakia (42%), and Eastern Slovakia (35.3%).

All results presented and discussed in this study were acquired through the survey that was conducted during summer 2020 following the first wave of the COVID-19 pandemic in Slovakia, and when pandemic measures and restrictions were eased. The survey was distributed digitally throughout the whole of Slovakia to all age, sex, and residence segments. The survey was carried out in collaboration with a market research agency Go4insight, who have expertise in qualitative and quantitative research and data collection methods. In this study, respondents were divided into four age groups (16–29, 30–44, 45–62, and >63 years of age), while sex consisted of two categories (female and male). Among other aspects, the survey consisted of 19 questions, including two questions asking respondents to provide their average NFV per month before and during the COVID-19 pandemic (Supplementary File S1). For the purpose of this study, we focused on 6 factors, i.e., sex, age, place of residence with regard to forest coverage (Figure 1), population size in place of residence, income category of respondents, and employment type. We then examined which factor had the most robust effect on NFV.

Statistical analysis was performed using R Statistical Software (Foundation for Statistical Computing, Vienna, Austria). In the first step, descriptive statistics were used to explore the data. In the second step, we analyzed the influence of sex, age, number of citizens, education, employment, salary, place of residence, and region on visiting of forests through ANOVA. Results were considered significant if *p* ≤ 0.05.

## 3. Results and Discussion

Average per capita NFV values showed an increasing tendency from 5.39 before to 5.87 after the introduction of pandemic measures (i.e., by 9%, *p* = 0.098). However, the rates of changes in NFV varied considerably in different population groups classified by geographical, socioeconomic, and demographic criteria.

### 3.1. Role of Forest Accessibility

**Figure 2:**
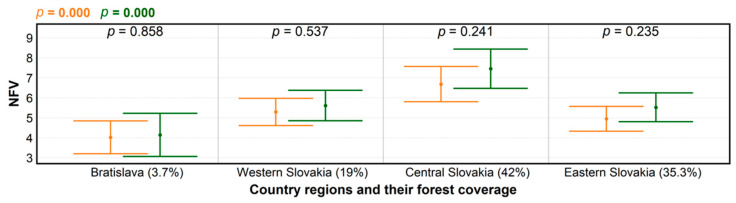
Levels of significance for differences in numbers of forest visits (NFV) per month according to regions characterized by forest coverage (%), before (orange) and during the COVID-19 pandemic (green), produced using ANOVA; and for NFV differences within groups (black), determined by *t*-test.

The ANOVA results pointed to strong regional influence on NFV, regardless of the pandemic’s first wave (Figure 2). We attributed this significant effect to distinct forest coverage, and thus also settlement distance to the nearest forest in each region. For example, the highest and the lowest average NFV differences occurred in the Central Slovakia and Bratislava regions, featuring the maximum and minimum forest coverage, respectively.

**Figure 3:**
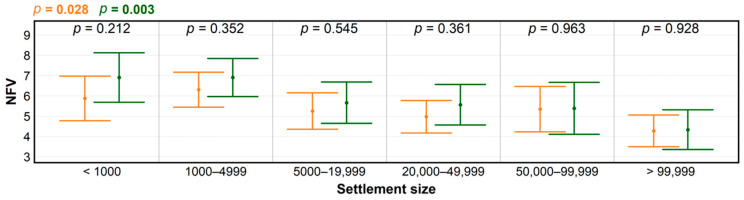
Levels of significance for differences in numbers of forest visits (NFV) according to settlement size, before (orange) and during the COVID-19 pandemic (green), produced using ANOVA; and for NFV differences within groups (black), determined by *t*-test.

It is very likely that the trend in NFV and its changes during COVID-19 with regard to forest coverage among Slovak regions resulted from a direct link between the forest coverage and distance to the nearest forest. Earlier studies conducted in some European countries showed that living closer to a forest increases the likelihood of greater frequency of forest visits, such as for children that grow up in the proximity of forests [23,24,25]. The proximity to forests was important, as people were prepared to walk to close recreational forests if the distance was less than 1–2 km. Otherwise they were likely to drive, but not necessarily to the nearest forest [26,27]. However, driving beyond one’s own district limits was severely restricted during COVID-19 lockdown. Thus, the effect of distance to the nearest forest helped explain the highest NFV increase in settlements with less than 1000 inhabitants, followed by the category of towns up to 5000 inhabitants (Figure 3). Both settlement types typically offer direct access to forests without the need to use motorized transport.

On the contrary, respondents from the most urbanized areas depend on public or private means of transport to reach forests. According to major inventories, half (52%) of all national forest visits were made by motorized visitors living within 100 km of national forest boundaries [28]. Similar figures were reported from Lithuanian forests [27]. However, the use of both public and private transport was either restricted, discouraged by authorities, or reduced by owners to avoid crowded parking lots during lockdown. Thus, on average, inhabitants of large cities without abundant recreational forests in their vicinity had fewer convenient opportunities for more frequent forest visits. In contrast, NFV probably spiked in conveniently located urban or periurban forests, e.g., a two-fold increase in NFV was reported from a forest near Bonn shortly after the inception of COVID-19-related measures [29]. Unlike average changes in NFV before and during the COVID-19 pandemic, such local or transient spikes were reported by Slovak media even in areas featuring low forest coverage, such as Bratislava region.

### 3.2. Roles of Income and Type of Employment

**Figure 4:**
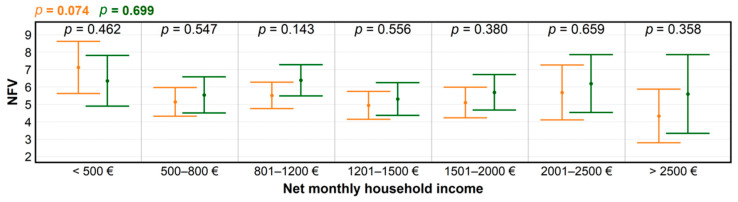
Levels of significance for differences in numbers of forest visits (NFV) among groups created according to net monthly household income, before (orange) and during the COVID-19 pandemic (green), produced using ANOVA; and for within-group NFV differences (black), determined by *t*-test.

**Figure 5:**
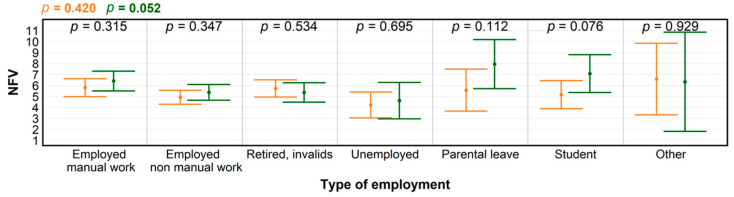
Levels of significance for differences in numbers of forest visits (NFV) according to type of employment, before (orange) and during the COVID-19 pandemic (green), produced by ANOVA; and for within-group NFV differences (black), determined by *t*-test.

Settlement size has been traditionally associated with average income, however this relationship appears to vary among countries [30,31]. Under normal circumstances, the shares of various income groups among nature and forest visitors tend to be rather similar [32]. The local visitors are more likely to come from lower household income groups than non-local visitors. However, the size of a settlement in Slovakia is not always a deciding factor affecting average income, and there are many exceptions from the rule due to multiple factors [33]. Regardless, COVID-19 related measures appear to have had an equalizing effect on average income–NFV relationships by cutting down and pushing up NFV in the lowest and the highest income categories, respectively (Figure 4). It is possible that the former group partly overlapped with the retiree category (Figure 5), for which the decrease was not significant. This indicates that the drop was partly offset by the increase of forest visitors from settlements with <1000 inhabitants (*p* = 0.22) in rural areas.

It also seems that flexible teleworking used by organizations to adapt to the global pandemic [34] enabled higher income categories to take advantage of increased flexibility to spend more time in nature, probably also with their children who did not attend schools at that time. 

Average income generally depends on the type of employment, which in turn became a more important factor with regard to NFV during the COVID-19 pandemic (*p* = 0.052). We deduced that the observed NFV increase in the majority of categories according to type of employment (Figure 5) resulted from the discovery of forests as environments providing opportunities for alternative leisure during widespread absence of established cultural and entertainment opportunities, and for coping with stress, building resilience, and inducing positive mood states in a time of increased anxiety and confinement [35,36,37]. The clearest manifestation of NFV increasing tendencies in some categories appeared for individuals on parental leave (*p* = 0.11). As for the “other” category, the highest NFV variability reflects its composition, including respondents from the IT sector, services, and state administration roles. While some individuals were placed on teleworking, others were included in the critical infrastructure, and thus had less control over their working schedules.

**Figure 6:**
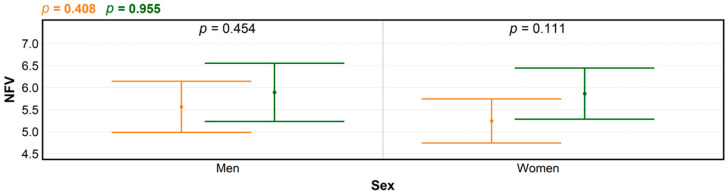
Levels of significance for differences in numbers of forest visits (NFV) between men and women, before (orange) and during the COVID-19 pandemic (green), produced using ANOVA; and for within-group NFV differences (black), determined by *t*-test.

The increases in NFV for the parental leave group before and after COVID-19 pandemic were clearly recognizable but statistically non-significant, and also did not lead to discernible NFV differences according to sex (Figure 6). While women still tend to stay home with young children despite the ongoing transformation of sex roles in European families [38], traditional role distribution could have been partially perturbed by the unexpected outbreak of COVID-19. Additionally, the respondent sample size did not specifically account for the parental leave category.

### 3.3. Role of Age

**Figure 7:**
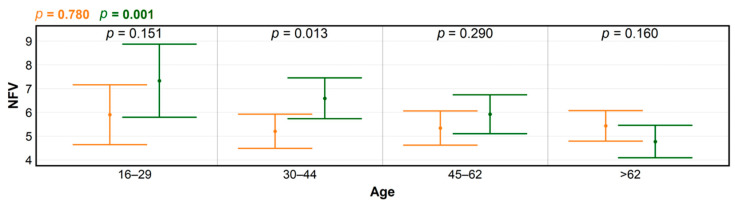
Levels of significance for differences in numbers of forest visits (NFV) according to age, before (orange) and during the COVID-19 pandemic (green), produced using ANOVA; and for within-group NFV differences (black), determined by *t*-test.

Finally, age as a factor explained a major part of the NFV variability (Figure 7). While there were no detectable differences in the numbers of visits according to age prior to the COVID-19 pandemic (*p* = 0.780), highly significant differences emerged during the event (*p* = 0.001). The largest increases—approximately 20% nominal increases in NFV—occurred for young and younger middle-aged individuals; the increase was also statistically significant for the latter category. In contrast, there was a notable decrease in the NFV rate for elderly people during the COVID-19 pandemic compared to the pre-COVID 19 level (*p* = 0.160), which corresponded with the nominal decrease in the “retirees and invalids” category (Figure 3). The different results in various age categories indicated that individuals had made differentiated risk assessments. For instance, visiting forests with high concentrations of visitors may have been perceived as a risk due to the increased probability of spreading or contracting COVID-19. In particular, national and local governments around the world have declared emergency measures, promoted stay-at-home orders, and required business closures to increase social distancing and reduce the risk of transmission [39]. In such contexts, it has been suggested that people generally make less risky decisions after a disaster [40]. However, other studies [41,42] have suggested a more nuanced and differentiated perspective, stressing that the risk attitudes of people who experience a disaster cannot be simply described as a general seeking or avoidance of risk, depending on whether information was gained by learning or by experience, age, and perception of low probability associated gain or loss. In our study, the main increase took place in young people and became less pronounced with increasing age, until there was a decrease in people older than 62 years. This corresponds well to theories implying that aging should be associated with reduced risk-taking. In fact, primarily older people were encouraged to “stay home” and “keep safe” as basic precautionary “shielding” measures promoted by health authorities and media outlets. The results also highlight possible vulnerabilities and disadvantages in certain population segments.

In fact, a plethora of factors and their interactions affected the numbers of visits during the COVID-19 pandemic, opening up avenues for further promising research. One question is whether the share of green urban spaces in municipalities affected NFV rates. We hypothesize that if people are aware of the utility of forest visits, as documented by an array of research outcomes, demand for accessible forest areas would increase despite available green city infrastructure.

## 4. Conclusions

The results from a nationwide survey of residents of Slovakia showed that forest accessibility was a paramount factor affecting the number of forest visits in both pre-COVID-19 situations and during the pandemic. In terms of the effects on NFV, settlement size was linked with forest accessibility through distance to the nearest forest, which was lowest in villages and towns with up to several thousand inhabitants. COVID-19 and its accompanying measures had an equalizing effect on average income–NFV relationships by alternatively diminishing and increasing NFV in the lowest and highest income categories, respectively. The observed pattern developed after widespread introduction of working-from-home schemes during the COVID-19 pandemic, accompanied by more flexible working schedules. Type of employment and age were revealed as additional crucial factors determining NFV rates. Additionally, there were nominal increases in stated NFV by respondents on parental leave and students on the one hand, while on the other hand NFV rates decreased in the retired people category, probably linked with risk avoidance behavioral patterns. It appears that COVID-19 triggered both conscious and unconscious responses by tapping into primordial and cultural connections between people, nature, and forests, as discussed by Schama [43]. As a result, both an intuitively and rationally propelled partial remedy to the constraints and stress caused by COVID-19 pandemic emerged in the form of an increase of NFV. It is crucial to maintain, adjust, and expand existing or design new opportunities for nature and forest recreation, especially in areas where such opportunities have been missing to date. Emphasis needs to be placed on protecting, enhancing, and building new nature area and forests with potential to provide restorative effects, considering and building on central pathways mediating the effects of nature and forests on human health. At the same time, the increase in NFV as a coping tool to handle health and wellbeing issues would not incur substantial public healthcare costs but could contribute significantly to enhanced public health. It is worth noting that the main challenges to enhancing wellbeing through forests are brought about by ecosystem and biodiversity degradation, deforestation, and climate change [4]. The findings from this paper imply that forest visits may be used as an effective tool to alleviate wellbeing issues in the face of perceived health risks. Therefore, a dialogue among forest owners, regulators, public health administrators, policy-makers, health officials, and all stakeholders should be facilitated to ensure the pursuit of a closer-to-nature, multiple-use-oriented land and forest management approach.

## Figures and Tables

**Table 1:** Determination of the respondent sample sizes. The required sample sizes were calculated for a 5% margin and 90% confidence level (CL). The realized sample sizes corresponded to the numbers of completed and returned questionnaires. Population size date were obtained from [22].

Variable	Stratum	Population Size	Required Sample Size	Realized Sample Size	Margin of Error (CL 90%)
Sex	male	2,194,802	271	470	3.79
	female	2,345,447	271	530	3.57
	total	4,540,249	271	1000	2.60
Age	16–29	868,926	271	107	7.95
	30–44	1,302,786	271	280	4.91
	45–62	1,317,266	271	276	4.95
	>62	1,051,271	271	337	4.48
Residence area	Bratislava (capital)	669,592	271	114	7.70
	Eastern Slovakia	1,627,704	271	299	4.76
	Central Slovakia	1,336,785	271	249	5.21
	Western Slovakia	1,823,792	271	338	4.47

CL—confidence level.

## Data Availability

The data presented in this study are available on request from the corresponding author.

## Supplementary Materials

Supplementary materials can be found at https://www.mdpi.com/article/10.3390/ijerph18073469/s1, File S1.
