# Supplementary material for: Variability in Forest Visit Numbers in Different Regions and Population Segments before and during the COVID-19 Pandemic
Source: Int J Environ Res Public Health. 2021 Mar 26;18(7):3469. doi: 10.3390/ijerph18073469 (PMC8037241; doi:10.3390/ijerph18073469)
Supplement: Supplementary file 1 [file ijerph-18-03469-s001.pdf]

Master Questionnaire
TU Zvolen
Project number: X209718

Length of interview:
Number of respondents: n1000/CAWI

SAMPLE VARIABLES

- Region (SC1) [S]
- Age (SC3) [O]
- Gender (SC4) [S]
- Size of the settlement (SC4) [S]
- Education (SC5) [S]

INTRODUCTION

We are currently conducting a survey on forest perception. We would appreciate your contribution. To begin with, please answer a few questions for statistical selection. Thank you in advance for your time.

DEMOGRAPHICS I

Base: all respondents

SC1. [S]
You are...
1. Male
2. Female

Base: all respondents

SC2. [Q]
How old are you?

Less than 16 years old
16 - 29 years old
30 - 44 years old
45 - 62 years old
63 years old and older

Base: all respondents

SC3. [S]
In which county do you live?

1. Bratislavský county
2. Trnavský county
3. Trenčiansky county
4. Nitriansky county
5. Žilinský county
6. Banskobystrický county
7. Prešovský county
8. Košický county

Base: all respondents

SC4. [S]
What is the population of the municipality / city in which you live?
1. up to 999 inhabitants
2. 1000 - 4999 inhabitants
3. 5000 - 19 999 inhabitants
4. 20 000 - 49 999 inhabitants
5. 50 000 - 99 999 inhabitants
6. 100 000 inhabitants and more

MAIN QUESTIONNAIRE

Base: all respondents
Q1. [M]

Base: all respondents
Q2. [S per row]

Base: all respondents
Q3. [S per row]

Base: all respondents
Q4. [M]

Base: all respondents
Q5. [poradie]

Base: all respondents
Q6. [S]

Base: all respondents
Q7. [Q]
On average, how many times a month did you visit the forest before the COVID-19 pandemic?

Base: all respondents
Q8. [Q]
On average, how many times a month did you visit the forest during the restrictions due to the COVID-19 pandemic?

Base: all respondents
Q9. [S per row]

STATISTICS
The following questions are for statistical purposes only.

Base: all respondents
SC5. [M]

Base: all respondents
SC6. [S]
You live in:

1. Detached family house
2. Terraced house
3. Apartment (block of flats, apartment building)
4. Other

Base: all respondents
SC7. [S]
What is your highest completed education?

1. Elementary
2. Apprenticeships, high school without GCSE
3. High school with graduation
4. University

Base: all respondents
SC8. [S]
What is your employment status?

1. Employed - manual work
2. Employed - non-manual work (sedentary work)
3. Pensioner, disabled pensioner

4. Unemployed
5. On maternity / parental leave / at home
6. Student (university and college/high school)
7. Other

Base: if employed SC8=1 or 2

SC9. [S]

Base: all respondents

SC10. [S]

To which category of net monthly income would you include your household? By this we mean the net monthly income of all members of the household. T*his data will be used solely for the statistical evaluation of the survey results.*

1. up to 500 EUR
2. 501 – 800 EUR
3. 801 – 1200 EUR
4. 1201 – 1500 EUR
5. 1501 – 2000 EUR
6. 2001 – 2500 EUR
7. more than 2500 EUR
